# Genome Sequence of a *Blattabacterium* Strain Isolated from the Viviparous Cockroach, *Diploptera punctata*

**DOI:** 10.1128/MRA.00229-20

**Published:** 2020-08-27

**Authors:** Emily C. Jennings, Matthew W. Korthauer, Joshua B. Benoit

**Affiliations:** a Department of Biological Sciences, University of Cincinnati, Cincinnati, Ohio, USA; Indiana University, Bloomington

## Abstract

Here, we report the genome sequence and characterization for a *Blattabacterium* strain isolated from the viviparous cockroach, *Diploptera punctata*, which provides amino acids critical for intrauterine embryo development. The genome was assembled by sequencing of the cockroach fat body, which is the location of this obligate symbiont.

## ANNOUNCEMENT

The Pacific beetle mimic cockroach, Diploptera punctata, reproduces by matrotrophic viviparity. D. punctata embryos develop inside the brood sac, a unique organ that functions as both a uterus and a pseudoplacenta; embryos are provided with nutrients by a secretion of milk-like components (1–5). The D. punctata milk is deficient in two essential amino acids, tryptophan and methionine (4, 6). It has been hypothesized that endosymbiont metabolism remediates this dietary deficiency; previous research suggests that blattabacteria are the exclusive component of the embryonic microbiome (7). We present a genome analysis of a *Blattabacterium* strain derived from D. punctata (*Blattabacterium* sp. strain DPU) to determine the potential role that this endosymbiont has during embryonic development of D. punctata.

Bacterial DNA was collected from fat body tissue dissected from a female D. punctata cockroach using a modified version of previously described protocols (8, 9) with the use of a Qiagen DNeasy Blood & Tissue kit. Samples were homogenized in 200 μl of sterile 1× phosphate-buffered saline. This extract was passed through a 20-μm glass syringe filter (Millipore) and centrifuged for 10 min at 8,000 × *g* at 4°C. The resulting pellet was resuspended in the extraction kit lysis buffer, and DNA was extracted following the manufacturer’s protocol. Illumina Nextera library preparation and HiSeq paired-end sequencing produced 6,778,349 paired-end reads of 75 bp and 4,444,306 reads of 125 bp. Less than 1% of reads were lost during quality control using Trimmomatic (10). metaSPAdes (v.1.2.2, with default settings) implemented in KBase (11, 12) generated 187 contigs with an *N*_50_ value of 625,590 bp, which is the length of the largest contig. BLASTn comparison of these contigs to those of the German cockroach *Blattabacterium* sp. strain Bbge genome (9) identified this largest contig as a candidate genome sequence (E value of <0.0001). Supported by subsequent BLASTn analyses against other *Blattabacterium* strains (E value of <0.0001), this contig was selected to be utilized as the genome sequence, and other contigs were discarded. BLASTn comparison of all metaSPAdes contigs to the Bbge plasmid (9) revealed that a 2,852-bp plasmid had been assembled as part of the genome; this sequence was removed from the contig for further analyses, producing a 623,008-bp contig with a GC content of 28.03% and 32.997× coverage. Coverage of the genome was assessed by mapping the paired-end reads to the extracted contig using Bowtie 2 (v.2.3.2) with default settings (13).

**FIG 1:**
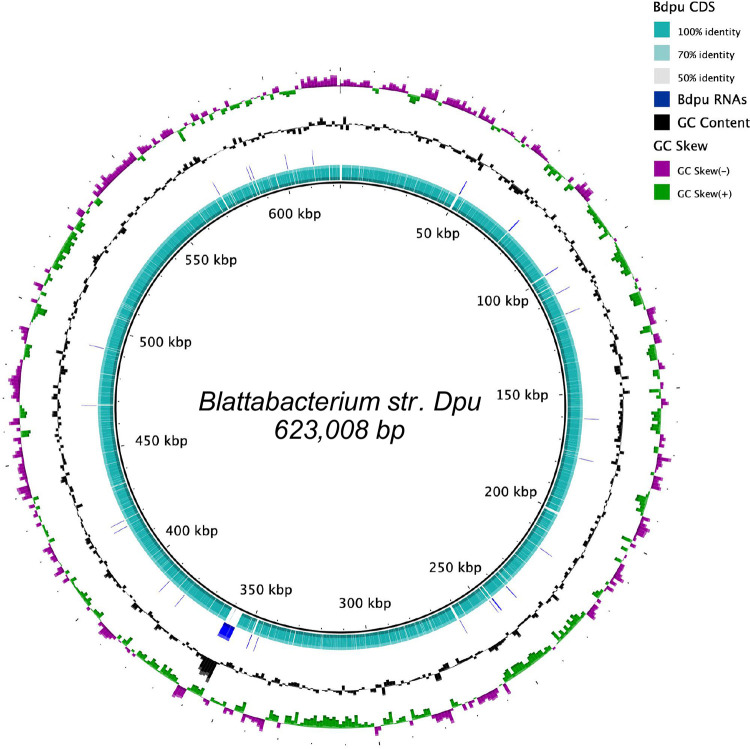
Genome presentation of *Blattabacterium* sp. strain DPU. Outer to inner rings represent GC skew (purple and green bars indicate negative and positive skew, respectively), GC content of each strand, RNA genes, including tRNA, rRNA, and transfer-messenger RNA genes (blue), and predicted coding sequences (teal). CDS, coding DNA sequence.

Prokka (14) identified 618 open reading frames, including 580 coding sequences, 34 tRNAs, 3 rRNAs, and 1 transfer-messenger RNA, using *Blattabacterium* with a similarity E value cutoff value of <0.0001. Almost all genes required for DNA replication, RNA transcription, and mRNA translational machinery were identified in the assembly (Fig. 1). dUTP nucleotidohydrolase, ribonucleoside diphosphate reductase subunit β, and two hypothetical proteins were identified in the plasmid. Orthology analysis using eggNOG-mapper (15) with the full available database revealed that most coding genes serve in translation and ribosome formation. The next most prominent known genome functions are amino acid metabolism and transport, followed by energy production and conversion. In addition to enzymes for central carbohydrate metabolism and nitrogen salvage, metabolic pathway prediction using the KEGG module mapper (16) identified complete biosynthetic pathways for nearly all essential amino acids. The traditional biosynthetic pathway for methionine is incomplete, however. Genes for all enzymatic reactions to produce methionine are present except for *metA*, which facilitates the conversion of homoserine and succinyl-coenzyme A to *O*-succinylhomoserine, and the alternative *metX*, which produces *O*-acetylhomoserine. An alternative methionine pathway has been suggested in other cockroaches (8), or shared synthesis could occur with the cockroach host based on genes identified in recent transcriptomic studies (5). However, the ability to synthesize selenomethionine is retained. *Blattabacterium* sp. strain DPU also has the ability to synthesize the nonessential amino acids alanine, arginine, cysteine, glutamate, and glycine.

### Data availability.

Illumina raw sequence reads and genome sequences have been deposited in association with BioProject PRJNA610624. The plasmid sequence has been deposited in GenBank under accession number MT645221.
